# Correlation between end-tidal carbon dioxide and the degree of compression of heart cavities measured by transthoracic echocardiography during cardiopulmonary resuscitation for out-of-hospital cardiac arrest

**DOI:** 10.1186/s13054-019-2607-2

**Published:** 2019-10-29

**Authors:** Roman Skulec, Petr Vojtisek, Vladimir Cerny

**Affiliations:** 1 Department of Anesthesiology, Perioperative Medicine and Intensive Care, J.E. Purkinje University, Masaryk Hospital Usti nad Labem, Socialni pece 3316/12A, 400 11 Usti nad Labem, Czech Republic; 2 Emergency Medical Service of the Central Bohemian Region, Vancurova 1544, 272 01 Kladno, Czech Republic; 3 Department of Anesthesiology and Intensive Care, Charles University, Faculty of Medicine in Hradec Kralove, University Hospital Hradec Kralove, Sokolska 581, 500 05 Hradec Kralove, Czech Republic; 4 Usti and Labem Region Emergency Medical Services, Socialni pece 799/7a, 400 11 Usti and Labem, Czech Republic; 5 Department of Research and Development, Charles University in Prague, Faculty of Medicine in Hradec Kralove, University Hospital Hradec Kralove, Sokolska 581, 500 05 Hradec Kralove, Czech Republic; 6 Department of Anesthesia, Pain Management and Perioperative Medicine, Dalhousie University, Halifax, Nova Scotia B3H 4R2 Canada

**Keywords:** End-tidal dioxide, Ultrasound, Hemodynamic-directed cardiopulmonary resuscitation

## Abstract

**Background:**

The concept of personalized cardiopulmonary resuscitation (CPR) requires a parameter that reflects its hemodynamic efficiency. While intra-arrest ultrasound is increasingly implemented into the advanced life support, we realized a pre-hospital clinical study to evaluate whether the degree of compression of the right ventricle (RV) and left ventricle (LV) induced by chest compressions during CPR for out-of-hospital cardiac arrest (OHCA) and measured by transthoracic echocardiography correlates with the levels of end-tidal carbon dioxide (EtCO_2_) measured at the time of echocardiographic investigation.

**Methods:**

Thirty consecutive patients resuscitated for OHCA were included in the study. Transthoracic echocardiography was performed from a subcostal view during ongoing chest compressions in all of them. This was repeated three times during CPR in each patient, and EtCO_2_ levels were registered. From each investigation, a video loop was recorded. Afterwards, maximal and minimal diameters of LV and RV were obtained from the recorded loops and the compression index of LV (LVCI) and RV (RVCI) was calculated as (maximal − minimal/maximal diameter) *×* 100. Maximal compression index (CImax) defined as the value of LVCI or RVCI, whichever was greater was also assessed. Correlations between EtCO_2_ and LVCI, RVCI, and CImax were expressed as Spearman’s correlation coefficient (*r*).

**Results:**

Evaluable echocardiographic records were found in 18 patients, and a total of 52 measurements of all parameters were obtained. Chest compressions induced significant compressions of all observed cardiac cavities (LVCI = 20.6 ± 13.8%, RVCI = 34.5 ± 21.6%, CImax = 37.4 ± 20.2%). We identified positive correlation of EtCO_2_ with LVCI (*r* = 0.672, *p* < 0.001) and RVCI (*r* = 0.778, *p* < 0.001). The strongest correlation was between EtCO2 and CImax (*r* = 0.859, *p* < 0.001). We identified that a CImax cut-off level of 17.35% predicted to reach an EtCO_2_ level > 20 mmHg with 100% sensitivity and specificity.

**Conclusions:**

Evaluable echocardiographic records were reached in most of the patients. EtCO_2_ positively correlated with all parameters under consideration, while the strongest correlation was found between CImax and EtCO_2_. Therefore, CImax is a candidate parameter for the guidance of hemodynamic-directed CPR.

**Trial registration:**

ClinicalTrial.gov, NCT03852225. Registered 21 February 2019 - Retrospectively registered.

## Background

There has been growing evidence that a strategy of personalized hemodynamic-directed cardiopulmonary resuscitation (CPR) may be a superior approach to conventional CPR based on a uniform approach for all patients [1]. Several measures may be used for this purpose [2]. However, all except end-tidal carbon dioxide are invasive parameters not suitable for implementation to pre-hospital CPR for out-of-hospital cardiac arrest (OHCA). This can limit further research and clinical implementation. Recently, intra-arrest ultrasound is becoming increasingly part of the CPR protocol and novel portable ultrasound devices allow sophisticated and reliable examination even in the field. It may help to detect potentially reversible causes of cardiac arrest and identify pseudo-pulseless electrical activity [3]. The other potential benefit from intra-arrest ultrasound is the assessment of quality of chest compressions by evaluating the degree of compression of the left and right ventricle. When using this mode, ultrasound could be another measure to guide hemodynamic-directed CPR. Therefore, we conducted a pre-hospital clinical study to evaluate whether the degree of compression of the right ventricle (RV) and the left ventricle (LV) induced by chest compressions during CPR for OHCA and measured by transthoracic echocardiography correlates with the levels of end-tidal carbon dioxide (EtCO_2_) levels measured at the time of echocardiographic investigation. We hypothesize that the degree of LV compression during CPR significantly correlates with EtCO_2_ with a correlation coefficient > 0.5.

## Methods

We carried out a prospective observational clinical study of adult patients resuscitated for out-of-hospital cardiac arrest. It was approved by the ethics committee (Ethics Committee, Masaryk Hospital Usti and Labem, Czech Republic, reference code 236/57). The study was conducted in accordance with the Helsinki Declaration and good clinical practice. Since it was an observational clinical study without any impact on the management of the patients and all patients were unconsciousness because of cardiac arrest, informed consent was not required. The study has been registered and assigned by trial registration number NCT03852225.

### Selection of participants

All patients included in the study were treated by the emergency medical services (EMS) of the Central Bohemian region due to the emergency calls for out-of-hospital cardiac arrest. This service is the exclusive provider of primary physician-based pre-hospital emergency care in the Central Bohemia Region, Czech Republic. The region includes both the rural and urban population, in total of 1,315,299 inhabitants on a total area of 11,015 km^2^. Participants were recruited from June 2018 to March 2019. Inclusion criteria were adult patients resuscitated by EMS for witnessed out-of-cardiac arrest of non-traumatic origin by physician EMS ambulance crew and availability of intra-arrest ultrasound investigation. Exclusion criteria were the presence of pericardial tamponade and the finding of collapsed right and/or left ventricle in the first intra-arrest ultrasound examination.

### Protocol

At the time of arrival of EMS ambulance with the presence of a physician in the crew, advanced life support following the recent guidelines has been initiated if it has not been already performed by a non-physician EMS crew present on the scene earlier [3]. After performing an initial rhythm assessment, shock delivery (if indicated), securing the airway by endotracheal intubation, providing mechanical ventilation and continuous chest compressions, the patients were assessed for compliance with inclusion criteria. If they did, baseline intra-arrest ultrasound examination was performed during the nearest rhythm check by TRACE (thoracic and abdominal sonography in cardiac arrest) protocol by a portable ultrasound device Vscan dual probe (GE Healthcare, Chicago, Illinois, USA) [4]. In the case of exclusion of cardiac tamponade and right and/or left ventricular collapse, the patients were enrolled in the study. After the enrollment, transthoracic echocardiographic investigation was performed during ongoing chest compressions in all of them. First, an imaging of the left and the right ventricle was performed from a subcostal four-chamber view. To minimize measurement error between measurements due to an unequal echocardiographic view, a sector probe was placed to the subcostal area at an angle of less than 45° to the abdominal wall to display the aortic bulb in addition to the atria and ventricles. Then, the probe was tilted up until the aortic bulb disappeared from the image. At this position, the investigated structures were recorded during at least three consecutive compressions in 2D mode and each loop was stored for further analysis. At the time of echocardiographic investigation, EtCO_2_ level was measured by a side stream technique (RespSense capnography monitor, Nonin Medical Inc., Plymouth, MN, USA). These examinations were repeated on each patient three times during advanced life support, at least 2 min apart. The examinations did not interfere with the course of advanced life support, and the results of echocardiographic imaging did not affect the ongoing resuscitation and were evaluated later. After termination of advanced life support, all data were recorded for each patient using the recent Utstein style guidelines [5].

### Echocardiographic calculations

**Fig. 1:**
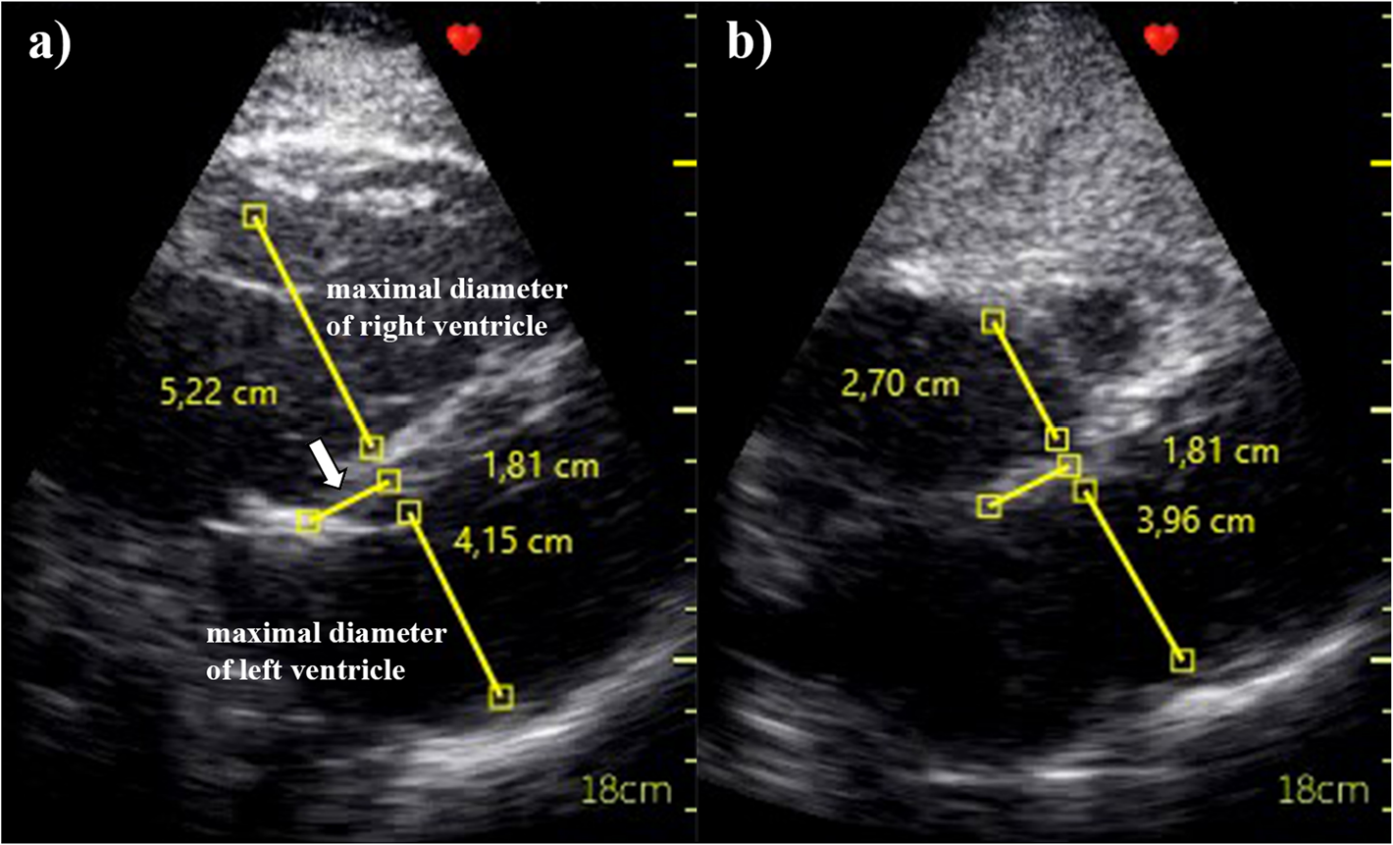
Measurement of the maximal and minimal diameters of the right and left ventricles for calculation of compression indexes. **a** The arrow indicates the distance of the measurement site from the mitral annulus. Measured values of maximal right and left ventricular diameters. **b** Measurements of the minimal diameters of the right and left ventricles at the same distance from the mitral annulus as the measurements at **a**

Maximal and minimal diameters of LV and RV were obtained from the recorded loops of 2D image (Fig. 1). For subsequent calculations, the cycle of compression and decompression was selected from an echocardiographic record, which allowed the most accurate measurement of the monitored parameters. In each measurement, the value of internal dimension of LV and RV was obtained perpendicular to the LV long axis and measured at or immediately below the level of the mitral valve leaflet tips [6]. A new parameter called a compression index (%) of LV (LVCI) and RV (RVCI) was calculated from each measurement as (maximal − minimal/maximal cavital diameter) × 100. Maximal compression index (CImax) was also calculated, defined as the value of LVCI or RVCI, whichever was greater at the time of measurement. Measurements of maximal and minimal diameter of both ventricles were performed by a single observer in a blinded manner. Ten randomly selected blinded loops were provided for measurement twice in order to calculate intra-observer variability. Echocardiographic records were also examined to determine if there was an evidence of complete mitral or tricuspid valve closure during compressions (Additional file 2).

### Statistical analysis

A sample size of 20 subjects was calculated for pilot study to identify a correlation of at least *r* = 0.5 between the degree of LV compression induced by CPR and levels of EtCO_2_. Mean values ± SD or percentages were calculated as necessary. Differences between groups were compared using the *χ*^2^ test, and statistical significance was calculated by the Fischer exact test for alternative variables. Statistical significance for continuous variables was determined by the paired Student *t* test. Spearman’s correlation coefficient was calculated for all correlations. Receiver operating characteristic (ROC) curve and Youden’s index were calculated for validity testing and identification of predictive thresholds. Relative intra-observer variability was expressed as absolute difference ± SD for maximal and minimal diameters of LV and RV. Data were analysed using Microsoft Excel 2010 (Microsoft, Redmond, WA, USA) and JMP 3.2 statistical software (SAS Institute, Cary, NC, USA). A *p* value of < 0.05 was considered statistically significant.

## Results

A total of 32 patients resuscitated for OHCA of non-traumatic origin were assessed for eligibility. Two of them were excluded because of the presence of exclusion criteria (collapse of the right and/or left ventricle found in baseline echocardiographic evaluation). A total of 30 patients underwent the study protocol. In 12 of them, echocardiographic records were not evaluable. Therefore, data from 18 patients were used for further analysis. In 16 patients, three echocardiographic measurements were performed before termination of cardiopulmonary resuscitation, in two patients only two. A total of 52 measurements of all parameters were obtained. Table 1 summarizes procedural and demographic data following the Utstein style of reporting. Table 2 shows relative intra-observer variability for measured echocardiographic parameters.

**Table 1:** Utstein style clinical characteristics of the patients

Number of the patients	18
Men/women (*n*)	13/5
Age (years ± SD)	66.6 ± 12.6
Location of OHCA (*n* (%))	
Home	12 (66.7)
Workplace	3 (16.7)
Street	1 (5.5)
EMS ambulance car	2 (11.1)
Aetiology of OHCA (*n* (%))	
Cardiac	11 (61.1)
Hypoxia	5 (27.8)
Pulmonary embolism	1 (5.5)
Metabolic	1 (5.5)
Witnessed OHCA (*n* (%))	18 (100.0)
First monitored rhythm (*n* (%))	
Ventricular fibrillation	6 (33.3)
Pulseless electrical activity	7 (38.9)
Asystole	5 (27.8)
Bystander CPR* (*n* (%))	
Compression and ventilation	2 (11.1)
Compressions only	10 (55.6)
Phone-assisted CPR** (*n* (%))	12 (66.7)
Time from collapse to any CPR attempt (s ± SD)	181 ± 160
Response time (s ± SD)	455 ± 292
BLS duration (s ± SD)	274 ± 264
ALS duration (s ± SD)	1916 ± 1085
Time from collapse to ROSC or CPR termination (s ± SD)	2371 ± 1210
Defibrillation time** (s ± SD)	467 ± 248
Any ROSC	12 (66.7)
Sustained ROSC	8 (44.4)
30-day survival or survival to discharge (*n* (%))	6 (33.3)
30-day favourable neurological outcome ((CPC score 1 or 2) (*n* (%))	5 (27.8)

*OHCA* out-of-hospital cardiac arrest, *EMS* emergency medical services, *CPR* cardiopulmonary resuscitation, *BLS* basic life support, *ALS* advanced life support, *ROSC* return of spontaneous circulation, *CPC* cerebral performance category

*In two patients with witnessed OHCA in the EMS ambulance car, BLS and phone-assisted CPR were not applicable. **If indicated

**Table 2:** Relative intra-observer variability for measurement of ventricular diameters from recorded echocardiographic loops

	Maximal diameter of the left ventricle	Minimal diameter of the left ventricle	Maximal diameter of the right ventricle	Minimal diameter of the right ventricle
Absolute difference (% ± SD)	1.10 ± 3.70	1,37 ± 2.2	2.01 ± 2.3	1.89 ± 2.3

Chest compressions induced significant compressions of both observed cardiac cavities (LVCI 20.6 ± 13.8%, RVCI 34.5 ± 21.6%, CImax 37.4 ± 20.2%). Table 3 demonstrates the values of compression indexes and EtCO_2_ levels in the separate measurements.

**Table 3:** Mean values of LVCI, RVCI, CImax and EtCO_2_ in separate measurements

	1st measurement	2nd measurement	3rd measurement
Number of patients (*n*)	18	18	16
Time from the beginning of ALS (s ± SD)	248.5 ± 85.2	529.5 ± 166.7	868.5 ± 232.5
EtCO_2_ (mmHg ± SD)	22.9 ± 4.1	23.6 ± 5.0	22.4 ± 7.3
LVCI (% ± SD)	21.8 ± 15.5	19.7 ± 13.3	20.3 ± 13.0
RVCI (% ± SD)	33.0 ± 21.2	36.8 ± 21.5	33.4 ± 23.3
CImax (% ± SD)	37.0 ± 19.6	40.0 ± 19.7	34.7 ± 22.0

*ALS* advanced life support, *EtCO*_*2*_ end-tidal carbon dioxide level, *LVCI* left ventricular compression index, *RVCI* right ventricular compression index, *CImax* maximal compression index

**Fig. 2:**
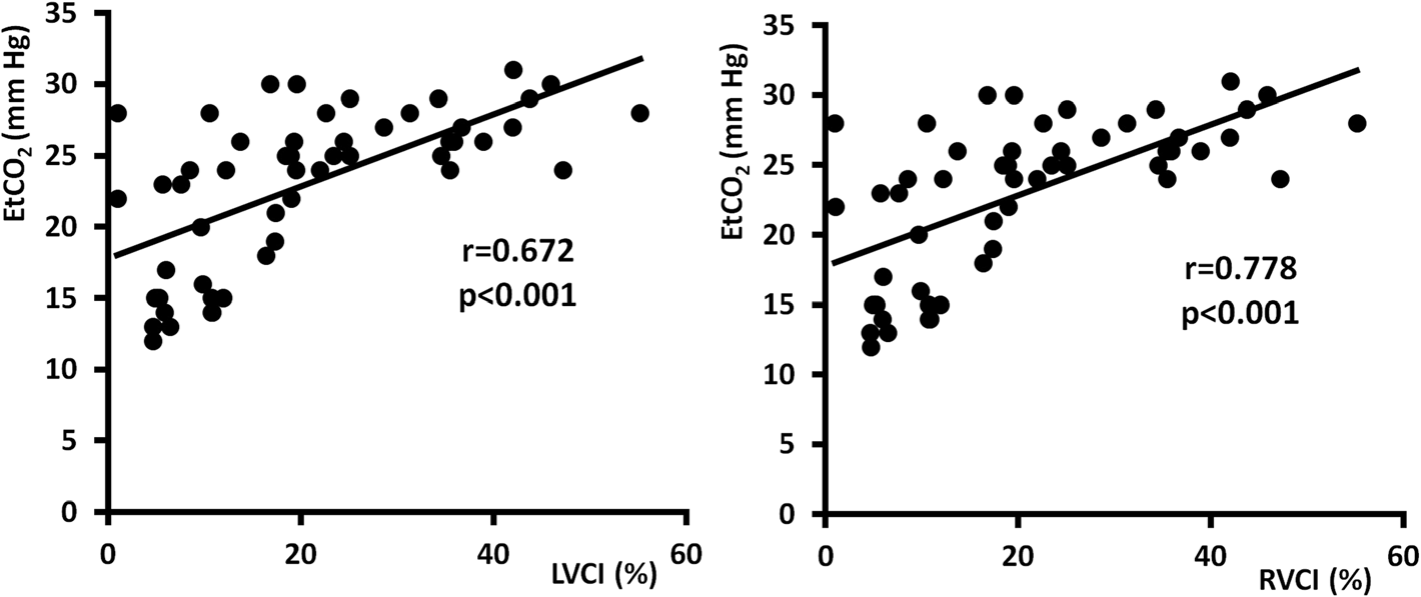
Correlation of EtCO_2_ with LVCI and RVCI. EtCO_2_—end-tidal carbon dioxide level, LVCI—left ventricular compression index, RVCI—right ventricular compression index

**Fig. 3:**
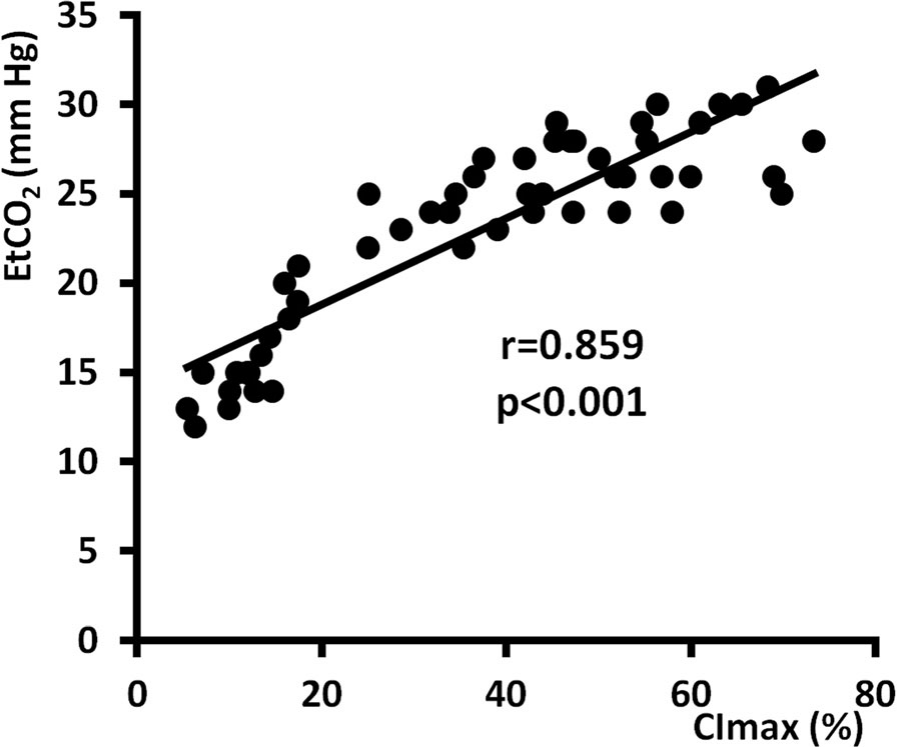
Correlation between EtCO_2_ and CImax. EtCO_2_—end-tidal carbon dioxide level, CImax—maximal compression index

**Fig. 4:**
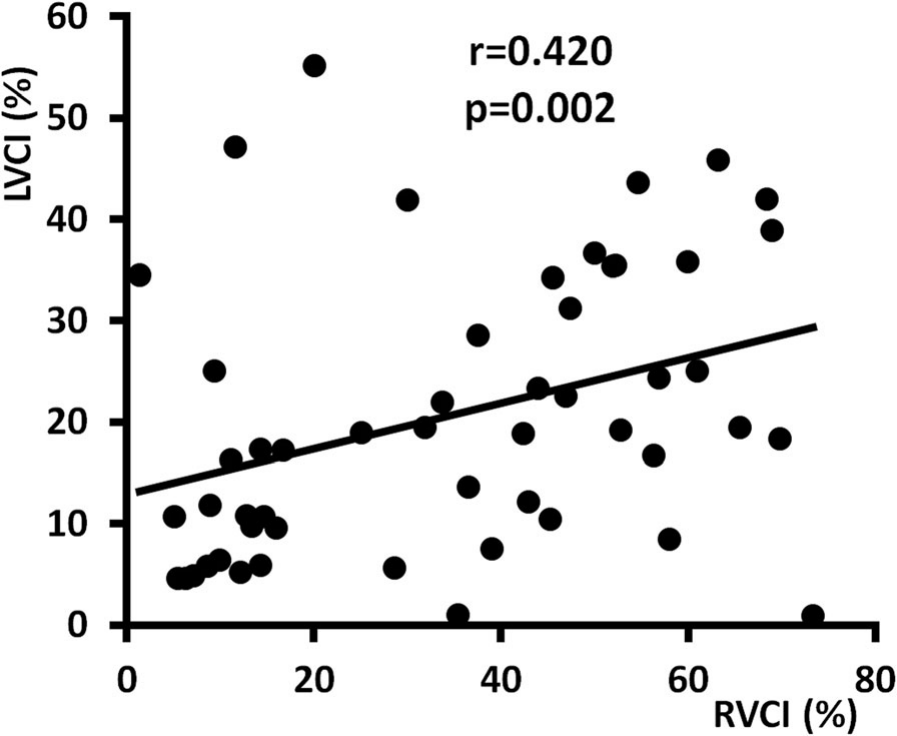
Correlation between RVCI and LVCI. LVCI—left ventricular compression index, RVCI—right ventricular compression index

**Fig. 5:**
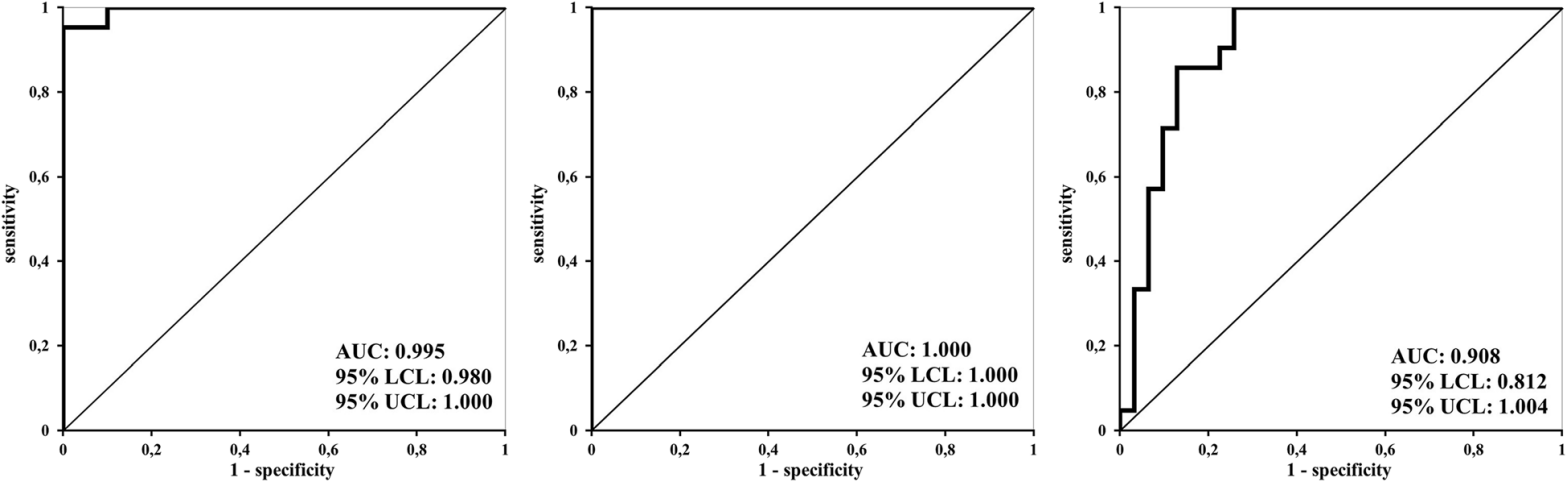
The ROC curves for prediction of different EtCO_2_ levels by CImax. On the left, prediction of EtCO_2_ > 15 mmHg, in the middle prediction of EtCO_2_ > 20 mmHg and on the right prediction of EtCO_2_ > 25 mmHg. EtCO_2_—end-tidal carbon dioxide level

Figure 2 demonstrates significant and clinically relevant correlation of EtCO_2_ with LVCI and RVCI. The strongest correlation was observed between EtCO_2_ and CImax (Fig. 3) (Additional file 1). On the other side, there was found a weak correlation between RVCI and LVCI (Fig. 4). Figure 5 shows the ROC curves for prediction of different EtCO_2_ levels by CImax. Table 4 describes cut-off levels of CImax for prediction of EtCO_2_ levels above different thresholds including Youden’s indexes. We identified that a CImax cut-off level of 17.35% predicted to reach an EtCO_2_ level > 20 mmHg with 100% sensitivity and specificity.

**Table 4:** CImax cut-off levels for prediction of EtCO_2_ levels above different thresholds

EtCO_2_ (mmHg)	CImax cut-off level (%)	Best Youden’s index	Sensitivity (%)	Specificity (%)	Positive predictive value	Negative predictive value
> 15	15.25	0.952	95.2	100.0	100.0	83.3
> 20	17.35	1.000	100.0	100.0	100.0	100.0
> 25	35.92	0.742	100.0	74.2	74.2	72.4

The values of the baseline echocardiographic parameters failed to predict any of the outcome measures. However, there was a significantly higher value of LVCI, RVCI and CImax in the third measurement in the patients with return of spontaneous circulation (ROSC) (LVCI 33.7 ± 8.1 vs. 12.3 ± 7.5%, < 0.001; RVCI 50.0 ± 10.5 vs. 23.4 ± 23.4%, 0.021; CImax 50.0 ± 10.5 vs. 25.6 ± 22.3%, 0.026) and 30-day/discharge survival (LVCI 35.6 ± 9.6 vs. 15.2 ± 9.7%, 0.002; RVCI 53.8 ± 10.5 vs. 26.6 ± 22.5%, 0.037; CImax 53.8 ± 10.5 vs. 28.4 ± 21.4%, 0.041) than in those who did not reached these outcomes.

In 24 measurements out of 52, we could identify the opening and complete closure of the mitral and/or tricuspid valve in synchronization with cardiac compressions. In 28 measurements, movement of valve leaflets was present, but due to insufficient image quality, it has not been possible to reliably claim that complete valve closure was present.

## Discussion

The main finding of the presented study is that a degree of compression of the left ventricle and the right ventricle evaluated by intra-arrest transthoracic echocardiography during ongoing chest compressions strongly correlated with the level of EtCO_2_ induced by CPR.

Performing advanced life support for out-of-hospital cardiac arrest should be carried out according to the detailed standardized protocol for advanced life support [3]. It is recommended that chest should be compressed to a depth of at least 5 cm but not more than 6 cm, chest compressions should be performed at a rate of 100–120/min and the hands should be placed on the lower half of the sternum [3]. However, there is a growing evidence that an anatomical relationship between the chest wall, sternal bone and the position of the heart in the chest is not uniform but may exert interindividual variability. It may be due to differences in normal chest configuration but also can be modified by morphological changes of intrathoracic organs because of heart, aortic or lung disease [7]. Functional pathophysiological changes of the chest, the heart and the lungs (e.g. lung compliance, cardiac diastolic dysfunction) can potentially affect the hemodynamic effect of standardized cardiopulmonary resuscitation as well [8].

Therefore, the transition from a conventional standardized CPR approach to personalized hemodynamic-directed CPR may be potentially useful to increase hemodynamic efficacy of CPR, ROSC rate and survival [1, 2]. The concept of hemodynamic-directed CPR requires several conditions: to have a parameter that reflects the hemodynamic efficiency of CPR; to have a knowledge of the target value of the parameter, which is associated with optimal hemodynamic efficiency of CPR; to have a protocol of modifying CPR that can achieve a goal and the parameter must be easily measurable in real time during CPR.

The concept of hemodynamic-directed CPR has been evaluated in several experimental studies. In swine models of induced cardiac arrest, targeting the CPR quality by manipulation with depth of chest compression and dose of vasopressors to predefined hemodynamic goal (systolic blood pressure, diastolic blood pressure, coronary perfusion pressure) resulted in increased brain tissue oxygenation, improved short- or long-term survival and better neurological outcome when compared with the standardized CPR approach following the recent guidelines [9–14]. There are also clinical data indicating that the concept of personalized CPR may be effective in clinical medicine. Berg et al. analysed prospective data from children resuscitated for in-hospital cardiac arrest. They found that diastolic blood pressure of ≥ 25 mmHg during CPR in infants and of ≥ 30 mmHg in children more than 1 year old was associated with higher chance of survival and favourable neurological outcome. Sainio et al. analysed data from 39 adult patients resuscitated for cardiac arrest with invasive blood pressure measurement and recording of CPR quality. They found that although the depth of compressions positively correlated with higher systolic and diastolic blood pressure, there was a significant heterogeneity in the relationship of depth of compressions and blood pressure. This finding strongly supports the idea of personalized hemodynamic-directed CPR [15].

However, the possibility of using invasive pressure monitoring during routine CPR for OHCA in the field is very limited. In the clinical setting of pre-hospital emergency medicine, measurement of EtCO_2_ may be beneficial. EtCO_2_ levels during CPR reflect pulmonary blood flow and cardiac output and may be used as potential non-invasive measure for personalized directing of CPR [16]. It has been shown that EtCO_2_ levels < 10 mmHg during CPR and especially after 20 min of CPR are associated with poor outcome while levels > 20 mmHg are likely to predict improved outcome, particularly ROSC [17–19]. It has been also demonstrated that EtCO_2_ levels measured during ongoing CPR reflect hemodynamic efficacy of CPR. Strong correlation of EtCO_2_ with cardiac output (*r* = 0.79, *p* < 0.001), coronary perfusion pressure (*r* = 0.78, *p* < 0.01) and cerebral blood flow (*r* = 0.64, *p* = 0.01) induced by CPR was observed [20–23]. In the observational clinical studies, Sandroni et al. and Murphy et al. analysed hundreds of patients resuscitated for cardiac arrest with synchronized EtCO_2_ measurement and continuous quality control of chest compressions. They found that an incremental increase of chest compression depth or chest compression rate was associated with a significant increase of EtCO_2_ levels [24, 25]. Therefore, although a clinical study evaluating the direct association of EtCO_2_ and cardiac output induced by CPR has not been published yet, experimental and clinical data support the idea that EtCO_2_ measurement may represent a valuable measure for hemodynamic-directed CPR.

In our pilot study, EtCO_2_ strongly positively correlated with the degree of compression of the right and the left ventricle. An important finding is that the correlation between LVCI and RVCI was weak and the strongest correlation was demonstrated between CImax and EtCO_2_. This suggests that in the setting of cardiac arrest and ongoing chest compressions, the heart works as a passive conduit divided by valves and it does not matter too much which ventricle we compress more, but the intensity of the compression of the heart is important. However, our clinical study does not provide a pathophysiological explanation for unequal compression of both ventricles and its large interindividual variability.

There are currently two main theories that explain the mechanism of induced blood flow during cardiopulmonary resuscitation, “cardiac pump theory” and “thoracic pump theory”. The question is whether measurement of LVCI, RVCI and CImax is applicable to both mechanisms or only to the “cardiac pump” mechanism. The first theory assumes that chest compressions directly compress the ventricles and, in conjunction with the mitral and tricuspid valve closure, generate forward blood flow. The second considers the rhythmic increase and decrease in intrathoracic pressure associated with each chest compression accompanied by induction of venous return and moving the blood from the heart to vessels. Mitral and tricuspid valves remain opened and retrograde blood flow is prevented by venous valves. This theory assumes collapse of the airways at each compression that allows an increase in intrathoracic pressure [26]. At present, it is not known which of the two is the main mechanism. Probably both may be involved, and the major factors that determine which mechanism will be dominant in the individual patient may be chest configuration, compliance of the chest wall, age, type of cardiac compression device (if used), force of the compressions and velocity of the compressions [26, 27]. These circumstances, especially the chest configuration, may be the reason for different mechanisms of generating blood flow by chest compressions in humans and experimental animals [28, 29]. In our study, we did not collect data to determine which mechanism of blood flow induction was involved in our patients. We were not able to reliably assess whether complete mitral and/or tricuspid valve closure was present during chest compressions in our patients due to insufficient recording quality at the level of the mitral and tricuspid annulus, we were unable to measure transmitral flow and we did not measure any direct parameter of cardiac output or blood flow induced by chest compressions. It might also seem that the determination of LVCI, RVCI and CImax is consistent only with the cardiac pump theory. Porter et al. examined 17 patients resuscitated for cardiac arrest by transoesophageal echocardiography. They observed complete mitral valve closure during compression (group I, cardiac pump mechanism) in 12, but not in 5 patients (group II, thoracic pump mechanism). Transmitral flow was present in all of them, in group I patients during the decompression phase and in group II patients during the compression phase. However, in all patients in both groups, measurable changes in the left and right ventricular size were present [29]. This means that rhythmic changes in ventricular size occur with both mechanisms of induced blood flow. Therefore, pragmatic echocardiographic monitoring of the degree of ventricular compression induced by CPR regardless of mitral valve function and blood flow mechanism may be useful to assess the hemodynamic efficiency of cardiac compressions.

Based on the above, our observation is a proof of the different effect of a standardized CPR on the heart and supports the concept of personalized CPR. Validity of CImax to predict EtCO_2_ exhibited excellent discriminatory value in our study, and cut-off value of CImax of 17.35% predicted EtCO_2_ with perfect sensitivity and specificity. Therefore, CImax is another candidate parameter for guidance of hemodynamic-directed CPR and for its non-invasivity, especially for the use in the field. However, there are several aspects that must be considered as potential limitations of its use. First, the question is whether compression indexes may be used for CPR guidance of cardiac arrest of all causes. In particular, the presence of right and/or left ventricular collapse (usually due to severe hypovolaemia) is associated with potential bias because of pre-existing marked alteration of preload and we assume that the values of compression indexes may be overestimated. A similar but opposite problem may occur with pulmonary embolism. Therefore, although these findings are crucial in the CPR setting, they were excluded from this pilot study. Second, the quality of transthoracic echocardiographic examination during ongoing cardiac compressions is not always sufficient for reliable measurement of dimensions of cardiac cavities. This was also the case in our study in 12 patients. This can be solved by using transoesophageal echocardiography in hospital. In the field, carefully finding the right subcostal probe position using aortic bulb disappearance as an anatomic landmark can increase validity and reproducibility of measurement. Third, a potential limitation of the compression index calculation during CPR is the need to measure the dimensions of the heart cavities and further calculation of the index immediately. For this purpose, it would be optimal to implement software for real-time automatic measurement and calculation into the ultrasound devices, and this is one of the topics we are working on.

### Study limitations

There are several limitations in our study. First, the presented group of patients is relatively small. However, it was a pilot study and measurements were repeated in individual patients. Second, another potential and more advanced way of data analysis would be to build a full hierarchical linear model with patients and measurements as categorical predictors and studied variable as a continuous covariate and their interactions. That design would allow us to study influence of all variables, automatically would consider correlations between variables and take into account that two or three measurements were always from one patient. However, the number of measurements was too small for this type of analysis, so we chose the method described above. Third, only physiological measure of estimation of cardiac output induced by CPR was EtCO_2_ measured by a traditional side stream technique. We did not measure any direct parameter of cardiac output or blood flow induced by chest compressions. This is a limitation, as discussed in more detail above. Fourth, in 12 patients out of 30 included, we did not receive evaluable echocardiographic records. This limitation of transthoracic examination was discussed above. Fifth, we do not exactly know whether compression indexes correlate with EtCO_2_ only when the “cardiac pump” mechanism is generating blood flow in an individual patient or the same applies also to the “thoracic pump”.

## Conclusions

In conclusion, quantification of the degree of compression of heart cavities induced by chest compressions during CPR for OHCA and measured by transthoracic echocardiography from subcostal approach is feasible. Compression indexes strongly correlate with the levels of EtCO_2_ measured at the time of echocardiographic investigation in our study. We consider it, especially CImax, to be promising non-invasive measures for ultrasonographic guidance of hemodynamic-directed CPR of the patients resuscitated for cardiac arrest. At the next steps, it is necessary to determine whether this parameter is applicable to all resuscitated patients or only to certain subgroups, what the target values are and what the optimal correction of chest compressions to reach the target is. This is also the goal of our further research.

## Supplementary information

**Additional file 1:** Scatterplot of EtCO2 and CImax presenting separate correlations between the data from the separate measurements. Additional data analysis showing separate correlations between EtCO2 and CImax separately from the first, the second and the third measurement. Correlations are very similar and support the consistency and reliability of the data in the Result chapter.

**Additional file 2:** Echocardiographic records of changes of left and right ventricular diameter during chest compressions in two different patients. The record from patient 1 shows marked compression of right ventricle induced by chest compressions that is substantially more intense than the left ventricular compression. The record from patient 2 demonstrates almost no compression of the right ventricle and more intense but still insufficient left ventricular compression.

## Data Availability

The datasets used and/or analysed during the current study are available from the corresponding author on reasonable request.

## Abbreviations

ALS: Advanced life support
BLS: Basic life support
CImax: Maximal compression index
CPC: Cerebral performance category
CPR: Cardiopulmonary resuscitation
EMS: Emergency Medical Services
EtCO_2_: End-tidal carbon dioxide
LV: Left ventricle
LVCI: Left ventricular compression index
OHCA: Out-of-hospital cardiac arrest
ROC: Receiver operating characteristic
ROSC: Return of spontaneous circulation
RV: Right ventricle
RVCI: Right ventricular compression index
TRACE: Thoracic and abdominal sonography in cardiac arrest

## References

[1] Marquez AM, Morgan RW, Ross CE, Berg RA, Sutton RM (2018). Physiology-directed cardiopulmonary resuscitation. Curr Opin Crit Care.

[2] Meaney PA, Bobrow BJ, Mancini ME, Christenson J, de Caen AR, Bhanji F (2013). Cardiopulmonary resuscitation quality: [corrected] improving cardiac resuscitation outcomes both inside and outside the hospital: a consensus statement from the American Heart Association. Circulation..

[3] Soar J, Nolan JP, Böttiger BW, Perkins GD, Lott C, Carli P (2015). European Resuscitation Council guidelines for resuscitation 2015. Section 3. Adult advanced life support. Resuscitation.

[4] Skulec R, Truhlar A, Knor J, Cerny V (2015). TRACE: a new protocol for ultrasound examination during out-of-hospital cardiac arrest. Resuscitation..

[5] Perkins GD, Jacobs IG, Nadkarni VM, Berg RA, Bhanji F, Biarent D (2015). Cardiac arrest and cardiopulmonary resuscitation outcome reports: update of the Utstein Resuscitation Registry Templates for out-of-hospital cardiac arrest. Resuscitation..

[6] Lang RM, Badano LP, Mor-Avi V, Afilalo J, Armstrong A, Ernande L (2015). Recommendations for cardiac chamber quantification by echocardiography in adults: an update from the American Society of Echocardiography and the European Association of Cardiovascular Imaging. J Am Soc Echocardiogr.

[7] Hwang K, Chon S-B, Im JG (2017). The optimum chest compression site with regard to heart failure demonstrated by computed tomography. Am J Emerg Med.

[8] Segal N, Robinson AE, Berger PS, Lick MC, Moore JC, Salverda BJ (2017). Chest compliance is altered by static compression and decompression as revealed by changes in anteroposterior chest height during CPR using the ResQPUMP in a human cadaver model. Resuscitation..

[9] Friess SH, Sutton RM, Bhalala U, Maltese MR, Naim MY, Bratinov G (2013). Hemodynamic directed cardiopulmonary resuscitation improves short-term survival from ventricular fibrillation cardiac arrest. Crit Care Med.

[10] Sutton RM, Friess SH, Maltese MR, Naim MY, Bratinov G, Weiland TR (2014). Hemodynamic-directed cardiopulmonary resuscitation during in-hospital cardiac arrest. Resuscitation..

[11] Morgan RW, Kilbaugh TJ, Shoap W, Bratinov G, Lin Y, Hsieh T-C (2017). A hemodynamic-directed approach to pediatric cardiopulmonary resuscitation (HD-CPR) improves survival. Resuscitation..

[12] Sutton RM, Friess SH, Bhalala U, Maltese MR, Naim MY, Bratinov G (2013). Hemodynamic directed CPR improves short-term survival from asphyxia-associated cardiac arrest. Resuscitation..

[13] Friess SH, Sutton RM, French B, Bhalala U, Maltese MR, Naim MY (2014). Hemodynamic directed CPR improves cerebral perfusion pressure and brain tissue oxygenation. Resuscitation..

[14] Lautz AJ, Morgan RW, Karlsson M, Mavroudis CD, Ko TS, Licht DJ (2019). Hemodynamic-directed cardiopulmonary resuscitation improves neurologic outcomes and mitochondrial function in the heart and brain. Crit Care Med.

[15] Sainio M, Hoppu S, Huhtala H, Eilevstjønn J, Olkkola KT, Tenhunen J (2015). Simultaneous beat-to-beat assessment of arterial blood pressure and quality of cardiopulmonary resuscitation in out-of-hospital and in-hospital settings. Resuscitation..

[16] Falk JL, Rackow EC, Weil MH (1988). End-tidal carbon dioxide concentration during cardiopulmonary resuscitation. N Engl J Med.

[17] Meaney PA, Bobrow BJ, Mancini ME, Christenson J, de Caen AR, Bhanji F (2013). Cardiopulmonary resuscitation quality: improving cardiac resuscitation outcomes both inside and outside the hospital. Circulation..

[18] Idris AH, Staples ED, O’Brien DJ, Melker RJ, Rush WJ, Del Duca KD (1994). End-tidal carbon dioxide during extremely low cardiac output. Ann Emerg Med.

[19] Paiva EF, Paxton JH, O’Neil BJ (2018). The use of end-tidal carbon dioxide (ETCO2) measurement to guide management of cardiac arrest: a systematic review. Resuscitation..

[20] Sanders AB, Atlas M, Ewy GA, Kern KB, Bragg S (1985). Expired PCO2 as an index of coronary perfusion pressure. Am J Emerg Med.

[21] Weil MH, Bisera J, Trevino RP, Rackow EC (1985). Cardiac output and end-tidal carbon dioxide. Crit Care Med.

[22] Lewis LM, Stothert J, Standeven J, Chandel B, Kurtz M, Fortney J (1992). Correlation of end-tidal CO2 to cerebral perfusion during CPR. Ann Emerg Med.

[23] Sandroni C, De Santis P, D’Arrigo S (2018). Capnography during cardiac arrest. Resuscitation..

[24] Murphy RA, Bobrow BJ, Spaite DW, Hu C, McDannold R, Vadeboncoeur TF (2016). Association between prehospital CPR quality and end-tidal carbon dioxide levels in out-of-hospital cardiac arrest. Prehospital Emerg Care.

[25] Sheak KR, Wiebe DJ, Leary M, Babaeizadeh S, Yuen TC, Zive D (2015). Quantitative relationship between end-tidal carbon dioxide and CPR quality during both in-hospital and out-of-hospital cardiac arrest. Resuscitation..

[26] Ewy GA (2018). The mechanism of blood flow during chest compressions for cardiac arrest is probably influenced by the patient’s chest configuration. Acute Med Surg.

[27] Feneley MP, Maier GW, Gaynor JW, Gall SA, Kisslo JA, Davis JW (1987). Sequence of mitral valve motion and transmitral blood flow during manual cardiopulmonary resuscitation in dogs. Circulation..

[28] Rudikoff MT, Maughan WL, Effron M, Freund P, Weisfeldt ML (1980). Mechanisms of blood flow during cardiopulmonary resuscitation. Circulation..

[29] Porter TR, Ornato JP, Guard CS, Roy VG, Burns CA, Nixon JV (1992). Transesophageal echocardiography to assess mitral valve function and flow during cardiopulmonary resuscitation. Am J Cardiol.

