# Supplementary material for: Correlation between end-tidal carbon dioxide and the degree of compression of heart cavities measured by transthoracic echocardiography during cardiopulmonary resuscitation for out-of-hospital cardiac arrest
Source: Crit Care. 2019 Oct 29;23:334. doi: 10.1186/s13054-019-2607-2 (PMC6819356; doi:10.1186/s13054-019-2607-2)

Scatterplot of EtCO2 and CImax presenting separate correlations between the data from the first, the second and the third measurement.

EtCO_2_… end-tidal carbon dioxide level, CImax…maximal compression index


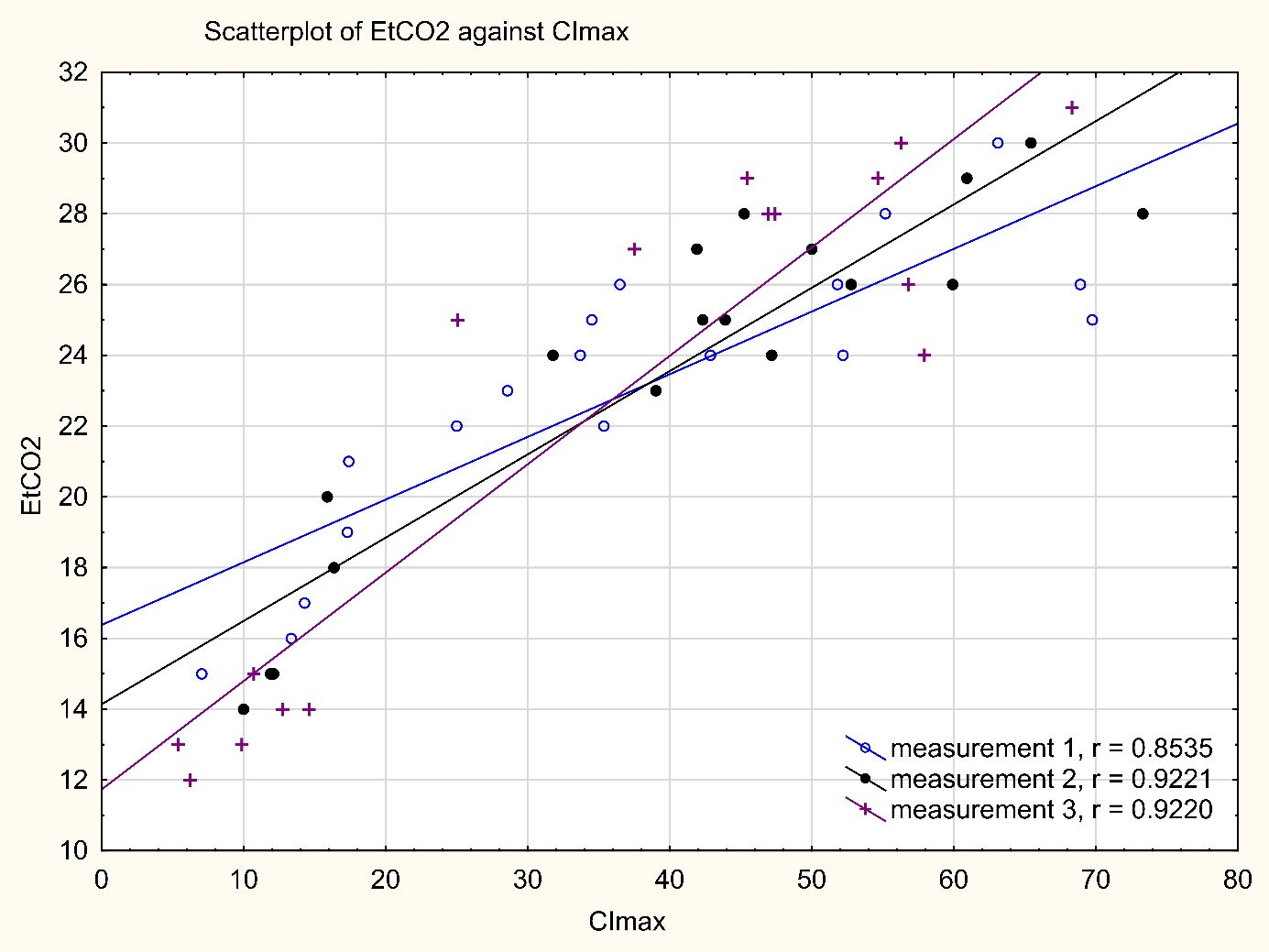

Supplement: Supplementary file 1 — Additional file 1: Scatterplot of EtCO2 and CImax presenting separate correlations between the data from the separate measurements. Additional data analysis showing separate correlations between EtCO2 and CImax separately from the first, the second and the third measurement. Correlations are very similar and support the consistency and reliability of the data in the Result chapter. [file 13054_2019_2607_MOESM1_ESM.docx]
